# Memristor Degradation Analysis Using Auxiliary Volt-Ampere Characteristics

**DOI:** 10.3390/mi13101691

**Published:** 2022-10-08

**Authors:** Georgy Teplov, Dmitry Zhevnenko, Fedor Meshchaninov, Vladislav Kozhevnikov, Pavel Sattarov, Sergey Kuznetsov, Alikhan Magomedrasulov, Oleg Telminov, Evgeny Gornev

**Affiliations:** 1 Laboratory for the Study of Neuromorphic Systems, Non-Volatile Memory Laboratory, Joint-Stock Company Molecular Electronics Research Institute, 124460 Moscow, Russia; 2 Industrial Artificial Intelligence, Artificial Intelligence Research Institute, 105064 Moscow, Russia; 3 Research Center in Artificial Intelligence in the Direction of Optimization of Management Decisions to Reduce Carbon Footprint, Skolkovo Institute of Science and Technology, 121205 Moscow, Russia

**Keywords:** memristor, silicon nitride, degradation, compact modeling

## Abstract

The memristor is one of the modern microelectronics key devices. Due to the nanometer scale and complex processes physic, the development of memristor state study approaches faces limitations of classical methods to observe the processes. We propose a new approach to investigate the degradation of six Ni/Si_3_N_4_/p+Si-based memristors up to their failure. The basis of the proposed idea is the joint analysis of resistance change curves with the volt-ampere characteristics registered by the auxiliary signal. The paper considers the existence of stable switching regions of the high-resistance state and their interpretation as stable states in which the device evolves. The stable regions’ volt-ampere characteristics were simulated using a compact mobility modification model and a first-presented target function to solve the optimization problem.

## 1. Introduction

The development of advanced microelectronic memory devices is related to the memristive effect [1]. The titanium oxide-based memristor [2] obtained in the Hewlett-Packard (HP) laboratory in 2008 demonstrated a promising direction [3], but its permissible switching frequency limited its applicability. Further research showed the possibility of obtaining the memristive effect using a variety of materials [4,5], some of which are applicable to existing technological processes.

One of the main drawbacks of most structures is the sample-to-sample characteristic instability and one sample operation instability [6]. The problem considered is observed in the memory window [7], which is the ratio of the resistances of the low-resistance (LRS) and high-resistance (HRS) states of the devices for the characteristic readout voltage. The evolution graph of this parameter describes the device degradation during device switching [8].

We developed six structures of bipolar memristor based on Ni/Si_3_N_4_/p+Si, relevant to modern microelectronic processes [9], and investigated the degradation process up to the device failure. The devices had a planar structure, and the Si_3_N_4_ layer was deposited with low-pressure chemical vapor deposition (LPCVD). The device retentions were up to $\sim 10^{4}$, and HRS to LRS resistance ratios constituted $10^{2}$. The performance structure investigation for neuromorphic tasks was out of the scope of the paper, but the characteristics of the devices correspond to the ones in relevant papers [9].

Silicon nitride was chosen as the active layer material for compatibility with Complementary Metal-Oxide-Semiconductor (CMOS) technology, physical properties of the material, including a high concentration of electron traps (≈$10^{19}\;{\mathrm{cm}}^{-3}$), high mechanical density, and chemical stability and availability of current models of silicon nitride-based memristor internal processes.

To study the degradation, we simulated the memristor operation in memory using fixed read and switch signals up to device failure. To analyze the device state, we proposed an additional recording of the volt-ampere characteristic (VAC), performed every 100 cycles. The obtained results were compared with earlier silicon nitride research in the Results and Discussion section (page 8), and other characteristics of the obtained samples were not investigated.

Moreover, we studied the fact that the registration of the VAC does not add qualitative distortions into the evolution curve of high resistance and may be used to study the stable switching between the memristor states.

To develop new approaches to simulate the device evolution and to estimate the influence of VAC measurements, switchings were performed for six memristors, with four interrupted by periodical VAC registration with an auxiliary signal. The obtained HRS curve and related VACs were used to investigate the evolution of the device state during its degradation.

The main contributions of this work are as follows:

- We developed six Ni/Si_3_N_4_/p+Si-based memristor structures and studied their degradation.
- We described two separate degradation processes of the memristive structure and proposed an approach to device state monitoring based on the auxiliary VAC registration.
- We proposed a new method to study the memristor degradation based on the resistance regions stable relative to switching resistance.
- We proposed a new target function for compact model parameter extraction and linked memristor degradation in stable regions with the VAC.
- We showed that the VACs from comparative stability regions of different samples are close to each other, which guarantees the generality of the proposed methods.

The rest of the paper is organized as follows. The Related Work section briefly provides the related research on evolution investigation approaches. The Materials and Methods section includes a description of the experiment setup, model equations, and used statistical methods. The Results and Discussion section includes the results of the experiments and model simulations. Finally, the Conclusions summarize our research and include possible feature work.

## 2. Related Work

Memristors are traditionally classified based on the conducting region type where resistance change occurs and electrode materials. The authors of [4] distinguish three main mechanisms: Filament formation, interface layer formation, and phase change switching. The change in the memristor state under the influence of the switching signal results in irreversible processes, the effect of which determines the peculiarities of the degradation process.

There are different types of degradation, e.g., with HRS increase or LRS increase or both at the same time [10,11]. However, the most frequent type is gradual HRS decrease and, as a consequence, the inability to switch from LRS to HRS [7].

In this paper, we investigated memristor structures based on the Ni/Si_3_N_4_/p+Si structure, which is relevant to modern microelectronic technology [9,12,13].

The switching mechanism in silicon nitride-based structures was studied in detail in [9,12,14]. In particular, in [9,14] the transport process is described by the model of space-charge-limited currents. The VAC analysis [9] allowed the authors to establish the filamentary nature of the switching mechanism.

The existence of stable relative to switching HRS resistance regions is associated by the authors with the preservation of some stable filament region relative to LRS-HRS transition, in accordance with the scheme of state change in the active layer proposed in [15].

The resulting memristors had two main degradation patterns [16]: Gradual reduction in the memory window with decreasing HRS and vice versa.

These switching processes are often simulated by compact models for memristor property and circuit studies [2,17,18]. The compact model design includes VAC parameter extraction [19] for achieving accuracy in structure behavior simulation under different signals.

In addition to the single VAC parameter extraction [19], modeling of memristor characteristic instability is being developed. There are two main methods—random sampling from a set of parameters extracted from multiple VACs and auxiliary state change equations based on the previous switching.

Memristor characteristic variation from cycle to cycle can be simulated with a number of approaches, such as time-series forecasting with autoregressive models [20] and a combination of memristor compact modeling and sampling [21,22,23,24]. In the second case, statistics for sampling are usually collected during parameter extraction from a set of volt-ampere characteristics. In some specific situations, current-voltage dependence could be data-driven, as well [24].

## 3. Materials and Methods

### 3.1. Experiment Description

The developed memristors had an MIS structure with a shared p-type silicon wafer as a substrate and bottom electrode, silicon nitride dielectric with a thickness of 4.5 nm, and nitride top electrodes. The insulating film is deposited on the entire surface of the bottom electrode. Top electrodes were patterned along the surface of the insulator.

**Figure 1:**
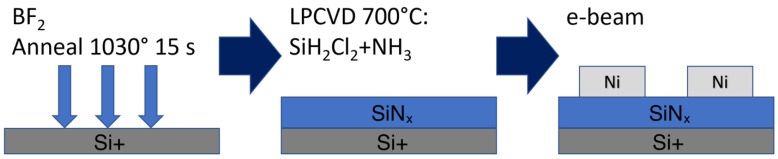
Technological process stages.

The technological process of producing the final Ni/Si_3_N_4_/p+Si structure includes several main stages (Figure 1). A p-type silicon substrate with a resistance of 12 Ω × cm^−2^ was used. Ion implantation (BF_2_^+^ with an energy of 40 keV and a dose of 10^15^ cm^−2^) was performed to form a highly doped p^+^ type layer in the substrate. After implantation, rapid thermal annealing was carried out at 1030 °C for 15 s. A 4.5 nm thick Si_3_N_4_ film was deposited by low-pressure chemical vapor deposition (LPCVD) at 700 °C, using a mixture of dichlorosilane (SiH_2_Cl_2_) and ammonia (NH_3_) in a ratio close to 1:3.5. Methods of metal deposition included e-beam deposition through a shadow mask. Six identical samples were produced for this study.

**Figure 2:**
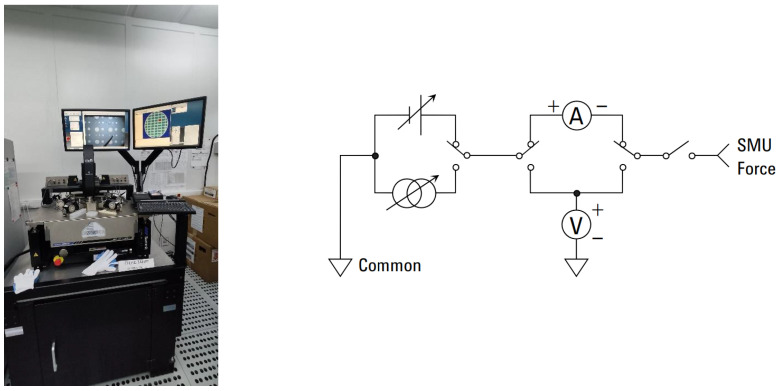
Experimental stand and simplified SMU circuit diagram.

The Cascade Microtech^®^ Summit 12k probe station with Agilent B1500A (Keysight Technologies; Beaverton, OR, USA) measuring equipment was used to measure the electrical parameters of the memristors. AC supplying was performed with the High Voltage Semiconductor Pulse Generator Unit (HV SPGU; Keysight Technologies; Beaverton, OR, USA) and current limiting by the High Power Source Measure Unit (HP SMU; Keysight Technologies; Beaverton, OR, USA). The reading of the current-voltage state was performed by the HP SMU. The DC waveform readout was performed by the HP SMU. The HP SMU device has a compliance feature that limits the output voltage or current to prevent damage to the device under test (Figure 2). The air temperature was 25 °C, the humidity was 48%, and the light was switched off during the experiment.

Instrumental errors of the equipment are presented in Table 1 and Table 2.

Signal pulse with an amplitude of 0.5 V switched the structure, and if the resulting resistance did not cross a certain threshold (5 kΩ for LRS and 50 kΩ for HRS), then a series (up to 20 attempts with 0.25 V amplitude increase per attempt) of additional switches were conducted.

As part of the development of new approaches to applying existing compact models to the simulation of device evolution, multiple volt-ampere recordings during switching were performed for four of the produced samples. Two more samples were used as reference samples to evaluate the influence of the measurements on the evolution process.

For the samples with VAC registration, for every 100th switching cycle by AC pulse, VAC registration of the DC switching process was performed. A series of 100 AC switching cycles and one DC switching cycle lasted until the breakdown. The breakdown criterion was a high-resistance state value less than 300 Ω.

**Figure 3:**
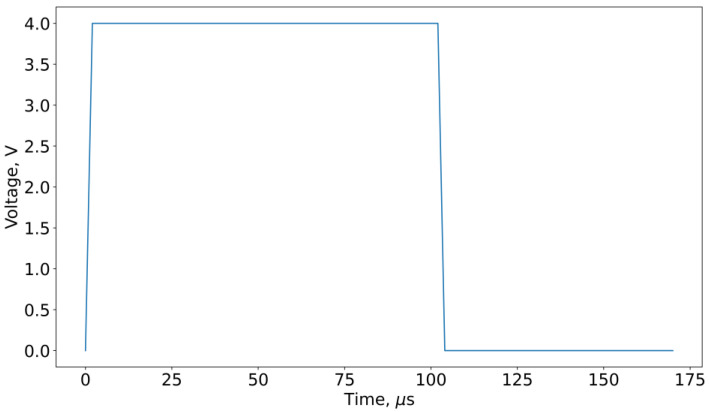
Characteristic waveform of SET pulse.

The typical form of pulses used in DC switching cycles is presented in Figure 3.

### 3.2. Degradation Processes Analysis

The degradation of a silicon nitride-based memristor is associated with structural changes in the high-resistance state. In general terms, we can distinguish two main mechanisms observed during the experiment—a smooth degradation with a decrease or increase in the resistance window, ending with an abrupt change in resistance, the direction of which is independent of the previous growth or decline of the curve.

**Figure 4:**
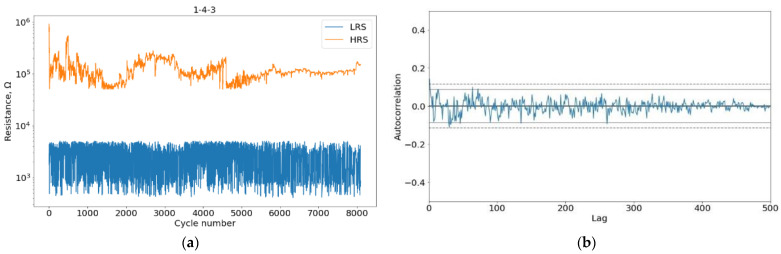
Example of HRS and LRS degradation (**a**) and autocorrelative function of LRS (**b**).

The evolution of the low-resistance state is weak for silicon nitride, and the associated LRS curve trends are insignificant against the high noise level. The LRS and its autocorrelation function are shown in Figure 4. For this reason, we did not use the LRS plot to study device degradation.

### 3.3. Stationary Regions of Degradation Processes

Regardless of the degradation process, observations can identify HRS regions that remain stable from switching to switching. That stability corresponds to the fact that switching after switching preserves some stable memristor structure of the high-resistance state.

Therefore, the memristor degradation process can be represented as a set of two process groups: Inside the stable states and the transition regions. It is necessary to consider the differences between stable states and transients to use this assumption in the degradation analysis.

One of the main assumptions is that trends in stable regions are more likely to correspond to the evolution of a single state, while abrupt changes correspond to a guaranteed state change.

Therefore, to determine the state stability conditions, we used resistance thresholds (±50% of the first resistance value in the tested region), the transition of which would correspond to a change in state and destruction of stationarity.

The second requirement is the definition of the minimum range at which we can say that the region is stable. Determining this size makes sense for researching several switches. Instead, we took a wide range of 100 switching cycles, which includes two registrations of the volt-ampere characteristic.

The proposed methods can be applied to a wide range of devices since they do not rely on specific physical mechanisms, such as the quantization of the memristor conductivity [25,26].

### 3.4. Structure VAC Registration as a Way to Analyze a Device State

The volt-ampere characteristic registration, which occurs in the middle of the stable region, does not destroy it by definition. Therefore, we can say that the obtained VAC form characterizes that stable region relative to the switching signal.

In this study, we produced six samples, and the evolution of two of them took place without recording the volt-ampere characteristics. We use these HRS curves as a reference to evaluate the VAC registration impact.

Therefore, we use the VAC curves to characterize the stability regions and consider device degradation as a process of state transition between these stability regions. As a result, the evolution of the volt-ampere characteristics can characterize the degradation of a memristor. Compact modeling is the primary method for extracting and using physical and mathematical parameters from the VAC curves.

### 3.5. Compact Modeling

In VAC analysis, the compact model of mobility modification [21], based on [27], was used. The model proposed involves simulation modeling of one volt-ampere characteristic. In the model, the existence of at least two ground states (LRS, HRS) is provided at the expense of the equation structure.

The current-voltage relation and evolution equation in this model take the following form:

(1) $$i=\left(\Pi_{i=1}^{n}U_{i}\left(x\right)\right)\{\begin{array}{cc} a_{1}x\sinh\left(bv\right), & v>0\\ a_{2}x\sinh\left(bv\right), & v<0 \end{array},$$

(2) $$\frac{dx}{dt}=g\left(v\right)f\left(x,v\right)$$

(3) $$g\left(v\right)=\{\begin{array}{cc} A_{p}\left(e^{v}-e^{V_{p}}\right), & v>V\\ -A_{n}\left(e^{-v}-e^{V_{n}}\right), & v<-V_{n}\\ 0, & -V_{n}\leq v\leq V_{p} \end{array},$$

(4) $$f(x,\;v>0)=\{\begin{array}{cc} e^{-\alpha_{p}\left(x-x_{p}\right)}w_{p}\left(x,x_{p}\right), & x\geq x_{p}\\ 1, & x<x_{p} \end{array},$$

(5) $$f\left(x,\;v\leq 0\right)=\{\begin{array}{cc} e^{-\alpha_{n}\left(x+x_{n}-1\right)}w_{n}\left(x,x_{n}\right), & x\leq 1-x_{n}\\ 1, & x>x_{n} \end{array},$$

(6) $$w_{p}\left(x,x_{p}\right)=\frac{x_{p}-x}{1-x_{p}}+1,$$

(7) $$w_{n}\left(x,\;x_{n}\right)=\frac{x}{1-x_{n}}.$$

where x is the state variable, $a_{1,2}$ and b are constants, $A_{p}$ and $A_{n}$ are constants that determine the change rate of the state variable after exceeding threshold voltages, $V_{p}$ and $V_{n}$ are the absolute values of the upper and lower threshold voltages, respectively. Parameters $x_{p}$ and $x_{n}$ are restricted only to the range [0, 1]. Parameter n is a hyperparameter representing the number of inhomogeneities taken into account, and functions $U_{i}\left(x\right)$ are the accounting functions of the inhomogeneities:

(8) $$U_{i}\left(x\right)=\{\begin{array}{cc} \exp\left(-\frac{{\left(x-x_{i}\right)}^{2}}{2\sigma_{i}^{2}}\right), & x<x_{i}\\ 1, & x\geq x_{i} \end{array}.$$

In Equation (8), $x_{i}$ is the effective position of the *i*-th inhomogeneity in the state space of a memristor, and $\sigma_{i}$ is the effective width of a given inhomogeneity.

### 3.6. Parameter Extraction Problem

We assumed that for silicon nitride, only the HRS curve may be used to describe and model degradation. Therefore, in VAC analysis the degradation should be observed via the analysis of VAC HRS branch. The most used way to extract compact model parameters involves the solution of an experimental VAC approximation problem via optimization.

The following new regularized target function was used to extract model parameters:

(9) $$L\left(P\right)=TF\left(P\right)+\lambda G\left(C\left(P\right),C_{0}\right)\to \underset{P}{min},$$

where P is a set of model parameters, λ is a regularization constant, C(P) is model contour, $C_{0}$ is approximated experimental contour, TF is the main part of the target function L, and G is a regularizator.

Here the main part is represented by the normalized symmetric difference area [21]:

(10) $$\mathrm{NSDA}=\frac{S\left(C\left(P\right)\Delta C_{0}\right)}{S\left(C_{0}\right)},$$

where $C\left(P\right)\Delta C_{0}$ is the symmetric difference in the regions bounded by the contours C(P) and $C_{0}$, and S(C) is the area of the region bounded by the experimental contour C.

To implement a more accurate modeling of HRS, MSE between HRS branches of experimental and model VACs was taken as a regularizer, with λ being equal to one.

## 4. Results and Discussion

**Figure 5:**
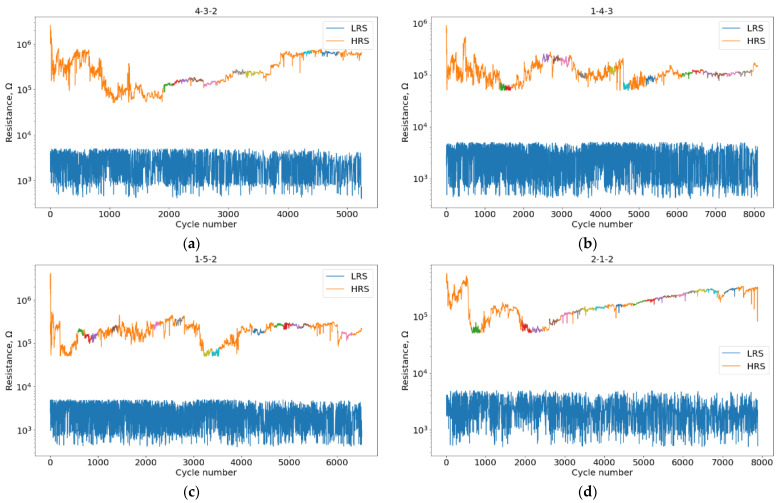
Plots of degradation with VAC registration, samples: (**a**) 4-3-2; (**b**) 1-4-3; (**c**) 1-5-2; (**d**) 2-1-2. Regions of stability on HRS are highlighted with colors different from orange.

**Figure 6:**
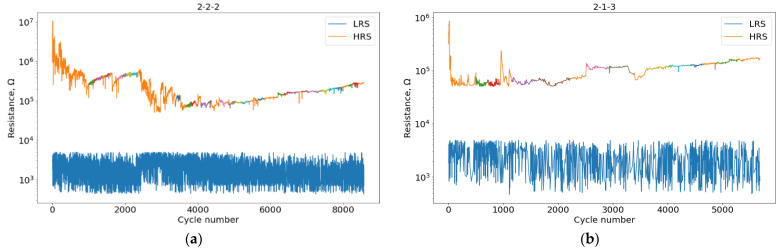
Plots of degradation without VAC registration, samples: (**a**) 2-2-2; (**b**) 2-1-3. Stability regions on HRS are highlighted with colors different from orange.

In the experiment described in the Experiment section, we obtained six samples (1-4-3, 1-5-2, 2-1-2, 2-1-3, 2-2-2, 4-3-2) with the presence and absence of VAC registrations. The switching resistance curves with the highlighted stability regions are shown in Figure 5 and Figure 6.

As discussed earlier, the state degradation plots represent a combination of two processes: A decrease or increase in the resistance of the HRS and a jump to another state. The combination of these processes was observed earlier [11,15] and general to all six samples. Failure of the device corresponds to an abrupt transition from the HRS and LRS resistances under 300 Ω.

**Figure 7:**
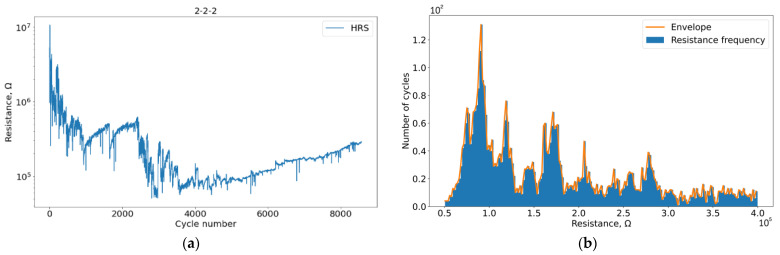
Plots for sample 2-2-2 without VAC registration: (**a**) Degradation of HSR during cycling; (**b**) HRS resistance histogram.

**Figure 8:**
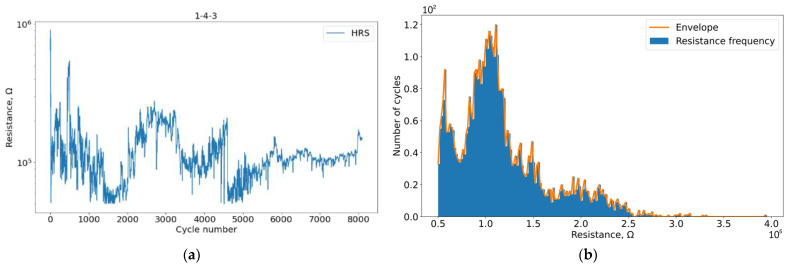
Plots for sample 1-4-3 with VAC registration: (**a**) Degradation of HSR during cycling; (**b**) HRS resistance histogram.

First, we investigated the effect of VAC registration on the evolution of the HRS curve. Due to the individual characteristics of each particular sample, the states obtained by histogram analysis (characteristic examples in Figure 7 and Figure 8) do not coincide exactly but are close enough to be correlated in the stability regions for samples in the presence and absence of VAC registration. 

**Figure 9:**
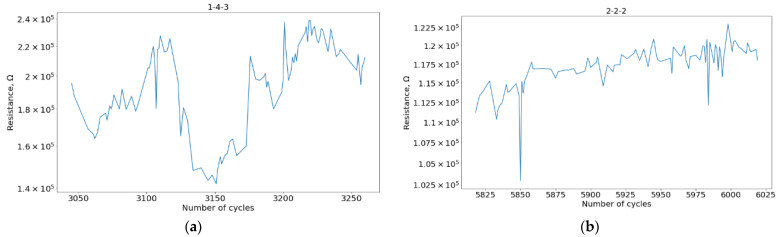
HRS curve example in the selected stability regions, devices: (**a**) 1-4-3 (with VAC registration); (**b**) 2-2-2 (without VAC registration).

A characteristic view of the stability regions estimated using the rule described in the Materials and Methods section is shown in Figure 9.

With the chosen approach to stability region selection, the VAC does not destroy stability and does not cause an additional variance, which could limit the overall nature of the conclusions drawn from the analysis of the VAC registration. The variance for the near-resistance states differs by 4.1% ($2.4\cdot 10^{12}$ and $2.3\cdot 10^{12}$) toward the graph without the VAC registration.

**Figure 10:**
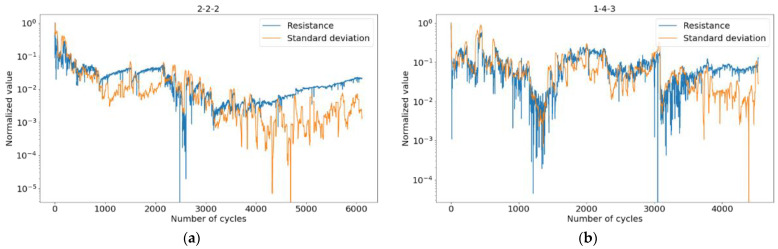
Resistance standard deviation for every 40 consecutive cycles, devices: (**a**) 2-2-2; (**b**) 1-4-3.

Within the framework of the proposed method, in addition to regions of smooth state degradation with low variance, the graphs show spikes and trends corresponding to transient states. We separated the areas using the standard deviation conditions on a preset number of 40 cycles (Figure 10), where transients correspond to large variance values.

**Figure 11:**
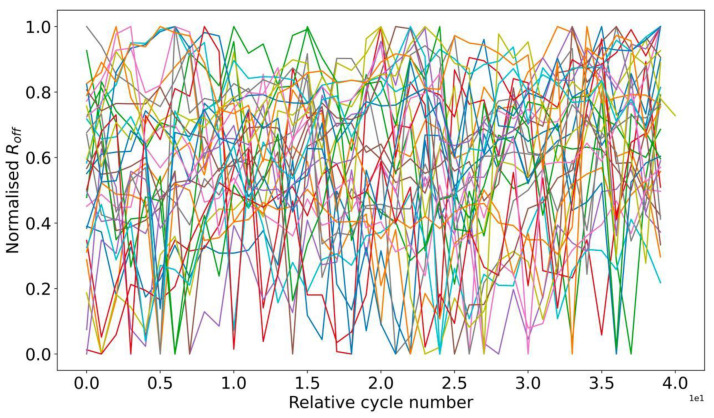
Normalized curve of 40 cycles after every registered VAC.

We checked whether the VAC registration may create some specific perturbations concerning the evolution of the HRS. Therefore, we plotted the curve behavior after VAC registration for the “2-1-2” sample in Figure 11.

**Figure 12:**
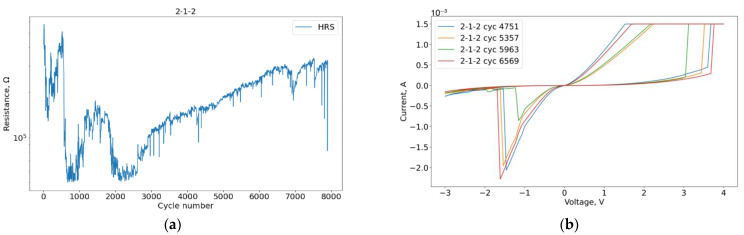
Plots for sample 2-1-2: (**a**) Degradation curve; (**b**) VACs in its stability region.

As a result, the effects of structure degradation are qualitatively independent of the registration of the I-V curve. Therefore, the preservation of stable resistance before and after its registration allows us to consider the VAC graph as characteristic of the current stable region of the device. The variation of the VAC for the stability areas is shown in Figure 12.

Due to Figure 12, the high resistant branch of the volt-ampere characteristic has the most significance for the analysis of device degradation processes. It was shown that despite the change in the characteristic switching voltage, the conductivity decreases monotonically along with the cycle number growth, preserving the order observed at the read voltage.

**Figure 13:**
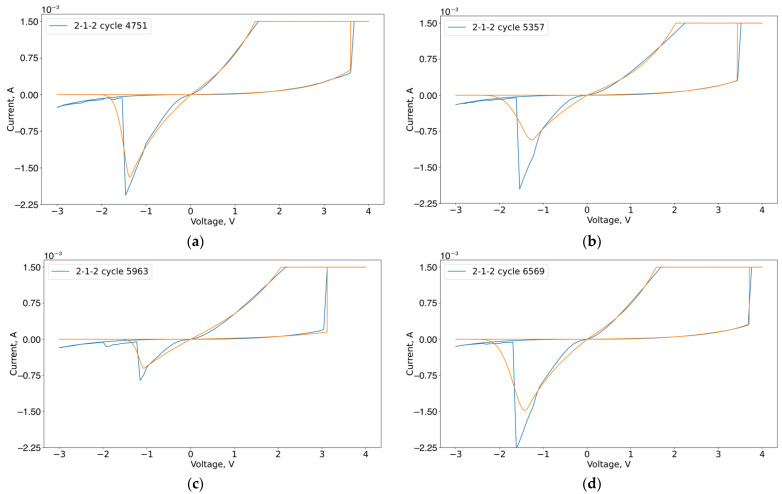
Approximation of experimental VACs of sample 2-1-2, cycles: (**a**) 4751; (**b**) 5357; (**c**) 5963; (**d**) 6569.

Next, we extracted the parameters of the previously considered compact model from the VACs for the stability and transient regions. The approximation results of the VACs by the presented compact model in the stable region are shown in Figure 13. Optimal parameters are in Appendix A, Table A1, Table A2, Table A3 and Table A4.

The following tendencies can be observed on the memristors considered: Threshold voltages have a positive or zero trend; the same behavior is inherent for parameters $a_{1}$ and $a_{2}$, which is responsible for the conductivity and x=1, and for evolution speed constants $A_{p}$ and $A_{n}$. The other parameters demonstrate zero or negligible trends.

The model commendable approximation of threshold characteristics as well as the whole VAC with some difference in the negative part of curves.

**Figure 14:**
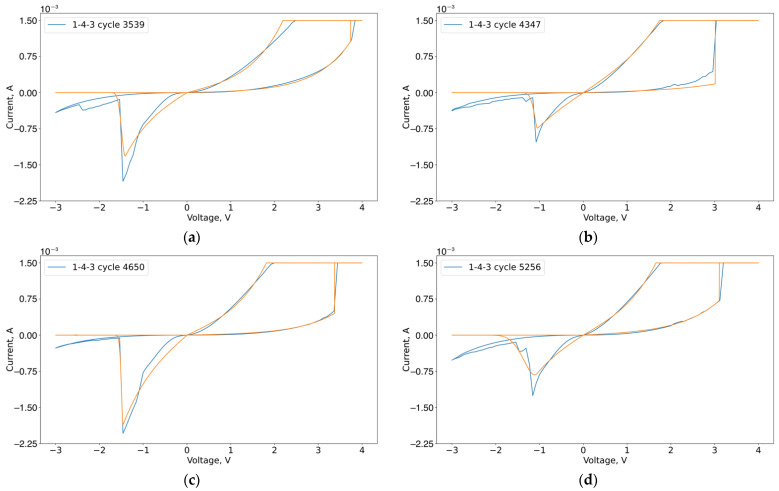
Approximation of experimental VACs of sample 1-4-3, cycles: (**a**) 3539; (**b**) 4347; (**c**) 4650; (**d**) 5256.

Optimal parameters for Figure 14 are in Table A5, Table A6, Table A7 and Table A8. The approximation of VACs in Figure 14 provides the following results: The only parameters with negative trends are $A_{n}$ and $V_{n}$. The others show a negligible trend. Qualitative VACs in the considered area differ from one another in the curvature of HRS and LRS branches as well as in threshold voltages.

The change in the threshold and switching voltages is independent of the degradation process and corresponds to the experimentally observed random occurrence of sticking, in which additional voltage is transferred to the device to change the state.

**Figure 15:**
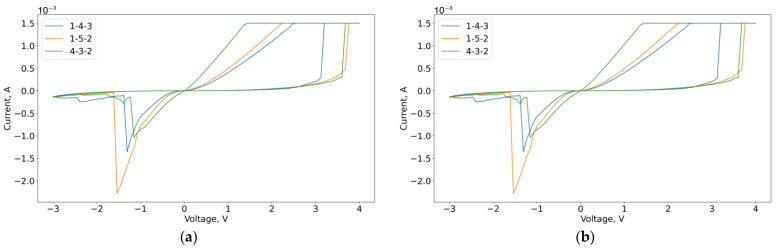
VACs with HRS corresponding to 120 kΩ (**a**) and 350 kΩ (**b**).

We evaluated the transferability of the approximation results, and for this purpose, we investigated the difference in the VAC curves between the samples. In this case, we considered the VACs for three samples, which have close resistance stability areas (120 kΩ), and the VACs near the same resistance (350 kΩ) from the transition region. The results are shown in Figure 15.

The results demonstrate that the VACs for close resistance stability regions not only replicate each other near the readout voltage but, in general, have the same shape with a number of quantitative local differences.

## 5. Conclusions

We designed and studied six samples of the actual Ni/Si3N4/p+Si-based bipolar memristor structure. For each of the samples, a study of the structure degradation was performed based on the analysis of a series of high and low device resistances resulting from the memory-like operations up to the failure of the memristor. We presented a new approach to device degradation analysis as an evolution of the memristor state between high-resistance stability regions and an approach to region localization.

We used the VACs in the stability regions to describe the memristor state and demonstrated that their registration does not qualitatively affect the resistance evolution. The VACs recorded in different samples with close stability regions are qualitatively close to each other, which allows for the extension of findings to other similar structures. We proposed a complex error function with accuracy enhancement on the high-resistance branch to investigate the degradation of a Ni/Si3N4/p+Si-based memristor using compact modeling.

Further work on developing the approach could be to find rules for predictive memristor model designing based on a stability region multi-samples study.

## Figures and Tables

**Table 1:** Instrumental errors, voltage.

Range	Force Resolution	Measurement Resolution	Force Accuracy ± (% + mV)	Measurement Accuracy ± (% + mV)
±20 V	1 mV	20 µV	±(0.018+3)	±(0.01+0.14)

**Table 2:** Instrumental errors, current.

Range	Force Resolution	Measurement Resolution	Force Accuracy ± (% + mV)	Measurement Accuracy ± (% + mV)
±1 nA	50 fA	10 fA	$\pm 0.1+3\cdot 10^{-13}+V_{0}\cdot \;10^{-15}$	$\pm 0.1+3\cdot 10^{-13}+V_{0}\cdot \;10^{-15}$

## Appendix A

**Table A1:** Optimal parameters for 4751 cycles of memristor #2-1-2.

Parameter	Value	Parameter	Value	Parameter	Value
$V_{p},\;V$	3.60	$a_{1}$, A	14.03	$x_{1}$	0.36
$V_{n},\;V$	1.34	$a_{2}$, A	17.89	$x_{2}$	0.56
$A_{p}$	2564	b, V^−^^1^	1.15	$x_{3}$	0.70
$A_{n}$	3458	$\sigma_{0}$	1.02	$x_{start}$	$1.40\cdot 10^{-3}$
$x_{p}$	0.02	$\sigma_{1}$	1.12		
$x_{n}$	0.72	$\sigma_{2}$	0.80		
$\alpha_{p}$	3267.66	$\sigma_{3}$	0.96		
$\alpha_{n}$	19.69	$x_{0}$	0.22		

**Table A2:** Optimal parameters for 5357 cycles of memristor #2-1-2.

Parameter	Value	Parameter	Value	Parameter	Value
$V_{p}$, V	3.12	$a_{1}$, A	12.69	$x_{1}$	0.38
$V_{n}$, V	1.20	$a_{2}$, A	14.27	$x_{2}$	0.44
$A_{p}$	3574	b, V^−^^1^	0.76	$x_{3}$	0.91
$A_{n}$	2682	$\sigma_{0}$	1.04	$x_{start}$	$2.58\cdot 10^{-3}$
$x_{p}$	$1.69\cdot 10^{-2}$	$\sigma_{1}$	1.90		
$x_{n}$	0.70	$\sigma_{2}$	1.16		
$\alpha_{p}$	2035.71	$\sigma_{3}$	0.87		
$\alpha_{n}$	21.65	$x_{0}$	0.18		

**Table A3:** Optimal parameters for 5963 cycles of memristor #2-1-2.

Parameter	Value	Parameter	Value	Parameter	Value
$V_{p}$, V	3.11	$a_{1}$, A	15.88	$x_{1}$	0.41
$V_{n}$, V	1.06	$a_{2}$, A	16.71	$x_{2}$	0.62
$A_{p}$	4429.68	b, V^−^^1^	0.80	$x_{3}$	0.85
$A_{n}$	3879.37	$\sigma_{0}$	1.01	$x_{start}$	$2.09\cdot 10^{-3}$
$x_{p}$	$2.08\cdot 10^{-2}$	$\sigma_{1}$	0.95		
$x_{n}$	0.75	$\sigma_{2}$	0.96		
$\alpha_{p}$	2061.91	$\sigma_{3}$	0.98		
$\alpha_{n}$	20.68	$x_{0}$	0.21		

**Table A4:** Optimal parameters for 6569 cycles of memristor #2-1-2.

Parameter	Value	Parameter	Value	Parameter	Value
$V_{p}$, V	3.71	$a_{1}$, A	20.69	$x_{1}$	0
$V_{n}$, V	1.35	$a_{2}$, A	25.75	$x_{2}$	0.39
$A_{p}$	7315.11	b, V^−^^1^	1.08	$x_{3}$	0.94
$A_{n}$	3681.71	$\sigma_{0}$	0.62	$x_{start}$	$9.90\cdot 10^{-4}$
$x_{p}$	$1.44\cdot 10^{-2}$	$\sigma_{1}$	0.69		
$x_{n}$	0.59	$\sigma_{2}$	2.03		
$\alpha_{p}$	1864.19	$\sigma_{3}$	0.68		
$\alpha_{n}$	15.79	$x_{0}$	3.40 e-2		

**Table A5:** Optimal parameters for 3539 cycles of memristor #1-4-3.

Parameter	Value	Parameter	Value	Parameter	Value
$V_{p}$, V	3.74	$a_{1}$, A	11.25	$x_{1}$	0.36
$V_{n}$, V	1.4	$a_{2}$, A	27.33	$x_{2}$	0.72
$A_{p}$	2537.74	b, V^−1^	1.26	$x_{3}$	0.87
$A_{n}$	21,726.15	$\sigma_{0}$	0.87	$x_{start}$	$3.60\cdot 10^{-3}$
$x_{p}$	0.02	$\sigma_{1}$	1.03		
$x_{n}$	0	$\sigma_{2}$	1.01		
$\alpha_{p}$	1441.76	$\sigma_{3}$	0.69		
$\alpha_{n}$	0.85	$x_{0}$	0.20		

**Table A6:** Optimal parameters for 4347 cycles of memristor #1-4-3.

Parameter	Value	Parameter	Value	Parameter	Value
$V_{p}$, V	3.01	$a_{1}$, A	16.31	$x_{1}$	0.39
$V_{n}$, V	1.04	$a_{2}$, A	16.61	$x_{2}$	0.60
$A_{p}$	4084.26	b, V^−1^	0.84	$x_{3}$	0.77
$A_{n}$	3898.61	$\sigma_{0}$	0.98	$x_{start}$	$2.04\cdot 10^{-3}$
$x_{p}$	$2.47\cdot 10^{-2}$	$\sigma_{1}$	0.99		
$x_{n}$	0.78	$\sigma_{2}$	1.07		
$\alpha_{p}$	2073.92	$\sigma_{3}$	1.02		
$\alpha_{n}$	19.79	$x_{0}$	0.20		

**Table A7:** Optimal parameters for 4650 cycles of memristor #1-4-3.

Parameter	Value	Parameter	Value	Parameter	Value
$V_{p}$, V	3.37	$a_{1}$, A	8.43	$x_{1}$	0
$V_{n}$, V	1.46	$a_{2}$, A	16.06	$x_{2}$	0.96
$A_{p}$	3721.70	b, V^−1^	1.17	$x_{3}$	0.94
$A_{n}$	5344.84	$\sigma_{0}$	0.52	$x_{start}$	$3.18\cdot 10^{-3}$
$x_{p}$	$3.10\cdot 10^{-2}$	$\sigma_{1}$	0.64		
$x_{n}$	0.74	$\sigma_{2}$	1.32		
$\alpha_{p}$	2615.70	$\sigma_{3}$	0.91		
$\alpha_{n}$	13.05	$x_{0}$	0.15		

**Table A8:** Optimal parameters for 5256 cycles of memristor #1-4-3.

Parameter	Value	Parameter	Value	Parameter	Value
$V_{p}$, V	3.10	$a_{1}$, A	15.73	$x_{1}$	0.39
$V_{n}$, V	1.05	$a_{2}$, A	17.71	$x_{2}$	0.63
$A_{p}$	1813.58	b, V^−1^	1.12	$x_{3}$	0.82
$A_{n}$	4086.36	$\sigma_{0}$	0.85	$x_{start}$	$3.46\cdot 10^{-3}$
$x_{p}$	0.015	$\sigma_{1}$	0.92		
$x_{n}$	0.69	$\sigma_{2}$	1.01		
$\alpha_{p}$	3421.10	$\sigma_{3}$	1.03		
$\alpha_{n}$	20.77	$x_{0}$	0.19		
